# VEGFR-dependent signaling contributes to postnatal brown adipose tissue development in Syrian hamsters

**DOI:** 10.1016/j.jphyss.2026.100106

**Published:** 2026-10-07

**Authors:** Khaled Mahmud Sujan, Yukino Tamakoshi, Bong Soo Seok, Zibo Yang, Shucheng Xu, Mohamed Elfeky, Mira Kato-Suzuki, Yuko Okamatsu-Ogura

**Affiliations:** a Laboratory of Biochemistry, Faculty of Veterinary Medicine, Hokkaido University, Sapporo, 060-0818, Japan; b Department of Physiology, Faculty of Veterinary Science, Bangladesh Agricultural University, Mymensingh, 2202, Bangladesh; c Department of Biochemistry, Faculty of Veterinary Medicine, Alexandria University, Alexandria, 21526, Egypt

**Keywords:** Angiogenesis, Brown adipose tissue, Cell proliferation, Sympathetic innervation, Syrian hamster

## Abstract

In most mammals, brown adipose tissue (BAT) forms before or soon after birth, whereas Syrian hamsters are born without BAT, which develops postnatally through the proliferation and differentiation of brown adipocyte progenitors. Since sympathetic innervation and vascularization are critical for BAT function, we examined their roles in postnatal BAT formation using interscapular adipose tissue from postnatal day (P) 6–14 hamsters. Putative progenitor-like cells were sparsely distributed at P6 and became progressively more abundant throughout the tissue with postnatal age. Sympathetic innervation emerged after P8, whereas blood vessels were detectable from P6 and increased with age during BAT development. Inhibition of vascular endothelial growth factor receptor (VEGFR) signaling by SU5416 significantly reduced cell proliferation and UCP1 expression. These findings suggest that VEGFR-dependent signaling contributes to postnatal BAT development in Syrian hamsters.

## Introduction

Mammals, including humans, have two types of adipose tissue: white adipose tissue (WAT) and brown adipose tissue (BAT). WAT serves mainly as an energy reservoir and is composed of white adipocytes characterized by a single, large lipid droplet and relatively few mitochondria. In contrast, BAT is specialized for non-shivering thermogenesis; brown adipocytes contain multiple small lipid droplets and a high density of mitochondria enriched in uncoupling protein 1 (UCP1), which enables the dissipation of energy as heat [1], [2], [3].

BAT formation involves the emergence of brown adipocyte progenitors from mesodermal precursors, followed by their proliferation and differentiation into UCP1-expressing adipocytes, accompanied by coordinated tissue remodelling to meet high metabolic demands [4], [5], [6]. Intrinsic transcriptional programs, including PRD1-BF1-RIZ1 homologous domain containing 16 (PRDM16) and bone morphogenetic protein (BMP) signaling, establish brown adipocyte identity [6], [7], [8], and extrinsic environmental stimuli, particularly temperature, play a critical role in regulating BAT mass and its functional maturation via stimulation of the sympathetic nervous system (SNS) [6], [9], [10].

The SNS serves as a central mediator that transmits environmental signals to regulate BAT activation and growth. Sympathetic innervation activates BAT through noradrenaline release and β-adrenergic signalling, stimulating thermogenic gene expression and promoting progenitor proliferation and adipocyte recruitment via cAMP-PKA pathways [9], [11], [12], [13]. In adult rodents, sympathetic activation is required for cold-induced BAT hyperplasia and adaptive expansion, as denervation markedly suppresses progenitor proliferation and BAT growth during cold exposure [11], [13]. Recent evidence further revealed that sympathetic fibers innervate BAT via both parenchymal fibers, which directly contact brown adipocytes, and vascular-associated fibers running along the arterioles to regulate blood flow and substrate delivery [14], [15], [16]. However, although the SNS is essential for BAT function in adults, its role in BAT development remains poorly understood.

In parallel with neural regulation, vascular development is tightly coupled to BAT growth. BAT is highly vascularized tissue that enables the efficient delivery of oxygen and metabolic substrates for thermogenesis [5], [17]. Cold exposure induces endothelial cell proliferation and vascular network expansion along with adipogenesis, highlighting coupled adipogenic-angiogenic remodelling during BAT expansion [10]. Vascular endothelial growth factor alpha (VEGFA) stimulates vascular remodelling and endothelial cell proliferation, and BAT‑specific VEGFA overexpression enhances vascularization, elevates Ucp1 expression, and improves thermogenic capacity [18], [19]. Moreover, perivascular regions harbor progenitor populations capable of giving rise to thermogenic adipocytes [20], suggesting that the vascular niche may actively influence adipocyte development. Notably, postnatal BAT development may share regulatory features with cold-induced BAT hyperplasia, including SNS activation, β-adrenergic signaling, progenitor proliferation, and angiogenesis [11], [13]. However, the specific contributions of sympathetic innervation and vascularization to early postnatal BAT formation remain incompletely defined.

The Syrian hamster provides a unique model to address these questions because, unlike most mammals, BAT is absent at birth and develops postnatally by the time of weaning [21], [22]. At birth, the interscapular depot is composed of white adipocytes that gradually convert into BAT. Thus, neonatal hamsters are functionally ectothermic and unable to maintain core body temperature until BAT formation is completed [23]. Our previous study demonstrated that during postnatal white-to-brown adipose tissue conversion, brown adipocyte progenitors emerge, proliferate, and subsequently differentiate into mature brown adipocytes, accompanied by active angiogenesis and endothelial proliferation [24]. In addition, rearing pups under warm conditions reduces sympathetic tone, decreases VEGFA expression and vascularization, suppresses progenitor proliferation, and diminishes UCP1-positive area in the interscapular depot [25], suggesting that both sympathetic activity and angiogenesis are associated with postnatal BAT development. However, because BAT formation proceeds even under warm conditions, it remains unclear whether sympathetic innervation and vascularization contribute to de novo BAT formation during postnatal development. In particular, it is unknown whether angiogenesis plays an active instructive role rather than serving merely as a permissive support system and whether sympathetic innervation plays a comparable role during this process. Therefore, the present study aimed to examine the temporal development of sympathetic innervation and vascularization and to investigate the contribution of VEGFR-dependent signaling to postnatal BAT formation in Syrian hamsters.

## Methods

### Animals and experimental procedures

All experimental procedures were approved by the Animal Care and Use Committee of Hokkaido University (Sapporo, Japan; approval nos. 18–0052 and 22–0116). Animals were maintained in a facility accredited by the Association for Assessment and Accreditation of Laboratory Animal Care International. Syrian hamsters (*Mesocricetus auratus*) were obtained from Japan SLC (Hamamatsu, Japan) and housed in plastic cages under controlled conditions (23 °C, 12:12-h light-dark cycle) with ad libitum access to standard laboratory chow (Oriental Yeast, Tokyo, Japan) and water. Females were housed with males for 1 week for mating. Pups of both sexes were used in the experiments.

For inhibition of vascular endothelial growth factor receptor (VEGFR), SU5416 (4 or 15 mg/kg; Sigma-Aldrich, St. Louis, MO), a selective VEGFR2 tyrosine kinase inhibitor, was administered intraperitoneally once daily from postnatal day 5 to day 9. SU5416 was dissolved in dimethyl sulfoxide (DMSO) at 20 mg/mL and stored at −20°C. Immediately before injection, the stock solution was diluted in vehicle containing 0.5% (wt/vol) carboxymethyl cellulose sodium (CMC-Na), 0.9% NaCl, 0.4% (vol/vol) Tween-80, and 0.9% benzyl alcohol. In separate experiments, the KIT inhibitor imatinib (10 or 25 mg/kg; MedChemExpress, Monmouth Junction, NJ, USA) or the MET inhibitor capmatinib (3 or 10 mg/kg; MedChemExpress) was administered intraperitoneally once daily from postnatal day 5 to day 9. Twenty-four hours after the final inhibitor injection, animals were euthanized by intraperitoneal administration of pentobarbital (Somnopentyl; Kyoritsu Seiyaku, Tokyo, Japan), and interscapular adipose tissue was collected for analysis. For age-dependent cell proliferation analysis, 5-ethynyl-2′-deoxyuridine (EdU; 50 mg/kg; Thermo Fisher Scientific) was administered intraperitoneally to pups aged 5–13 days and interscapular adipose tissue was collected 24 h after EdU administration.

For assessment of proliferation under VEGFR inhibition, EdU (50 mg/kg) was administered intraperitoneally at the time of the final SU5416 injection, and tissue was collected 24 h later. In all experiments, animals were euthanized by intraperitoneal administration of pentobarbital prior to tissue collection.

### Histological and immunofluorescence analysis

Tissue specimens were fixed in 4% paraformaldehyde, paraffin-embedded according to standard procedures, and cut into 4-µm-thickness. The sections were stained with hematoxylin and eosin (H&E) for morphological evaluation.

For immunofluorescence (IF) staining, sections were deparaffinized, rehydrated, and permeabilized with 0.3% Triton X-100 for 1 h, followed by washing with phosphate-buffered saline (PBS). Non-specific binding was blocked using Blocking One (Nacalai Tesque, Kyoto, Japan) for 10 min at room temperature and sections were incubated overnight at 4°C with primary antibodies diluted in PBS: anti-monocarboxylate transporter 1 (MCT1; Merck Millipore, Billerica, MA; 1:200), anti-tyrosine hydroxylase (TH; Sigma-Aldrich, St. Louis, MO; 1:250), or biotin-labeled *Maackia amurensis* agglutinin lectin (MAM-lectin; J-Chemical, Tokyo, Japan; 1:250). For multicolor staining, primary antibodies were applied simultaneously. After washing, sections were incubated for 1 h at room temperature with secondary reagents: Alexa Fluor 488-conjugated anti-chicken IgG, Alexa Fluor 647-conjugated anti-rabbit IgG (Thermo Fisher Scientific), or Alexa Fluor 594-conjugated streptavidin (BioLegend, San Diego, CA). Slides were mounted using ProLong Gold Antifade Mountant containing DAPI (Thermo Fisher Scientific) and visualized with a confocal microscope (LSM800; Zeiss, Oberkochen, Germany). Quantitative analysis was performed using the ImageJ software (National Institutes of Health, Bethesda, MD, USA) by averaging the values of at least three representative fields per specimen.

Cell proliferation was assessed using the Click-iT™ EdU Imaging Kit with Alexa Fluor™ 488 (Invitrogen, Carlsbad, CA, USA). Following deparaffinization, rehydration, and permeabilization, as described above, sections were blocked with Blocking One for 10 min. EdU incorporation was detected according to the manufacturer’s instructions by incubating sections with the reaction cocktail for 30 min at room temperature. For co-staining experiments, IF was subsequently performed as described above.

### Real-time PCR

Total RNA was extracted from adipose tissues using RNAiso Plus (Takara Bio, Shiga, Japan) in accordance with the manufacturer’s protocol. RNA was reverse transcribed using a 15-mer oligo(dT) adaptor primer and M-MLV reverse transcriptase (Promega, Madison, WI, USA). Quantitative real-time PCR was performed using a LightCycler® fluorescence thermal cycler (Roche, Mannheim, Germany) with Brilliant III Ultra-Fast SYBR Green QPCR Master Mix (Agilent Technologies, Santa Clara, CA, USA). Relative mRNA expression levels were calculated using the comparative cycle threshold (∆∆Ct) method and normalized to β-actin *(Actb)*. Actb was selected as the housekeeping gene after evaluating several candidate reference genes in Syrian hamster BAT and has also been used in our previous studies [24], [25]. The primer sequences are listed in Table 1.

**Table 1:** Primers used in Real-Time PCR.

Gene Name (Symbol)	Primer	Sequence 5′–3′
Uncoupling protein 1 *(Ucp1)*	Forward	GAG CTG GTA ACA TAT GAC CT
	Reverse	TGT CCT GGC AGA GAG TTG AT
Monocarboxylate transporter 1 *(Mct1)*	Forward	TCC AGT AAT GAT CGC TGG TG
	Reverse	TTA AGT GGA GCC AGG GTA GA
Vascular endothelial growth factor alpha *(Vegfa)*	Forward	ATC ACC ATG CAG ATC ATG CG
	Reverse	TTG CAA CGC CAG TCT GTG TT
Platelet endothelial cell adhesion molecule 1 *(Pecam1)*	Forward	GAG GTC ATA AAA GGA CAT GC
	Reverse	GTG GGC TTA TCT GTG AAT GT
ß-Actin *(Actb)*	Forward	AAG TGT GAC GTT GAC ATC CG
	Reverse	GAT CCA CAC AGA GTA CTT GC

### Western blotting

Interscapular adipose tissue was homogenized in ice-cold Tris-ethylenediaminetetraacetic acid (EDTA) buffer (10 mM Tris and 1 mM EDTA, pH 7.4) containing phosphatase (Nacalai Tesque, Kyoto, Japan) and protease inhibitor cocktails (Sigma-Aldrich, St. Louis, MO, USA). The homogenates were centrifuged at 800 × g for 10 min at 4°C, and the supernatants were collected as total protein extracts. In brief, protein was separated by SDS-polyacrylamide gel electrophoresis and transferred onto polyvinylidene fluoride membrane (Immobilon; Millipore, Tokyo, Japan). After blocking with 5% skimmed milk (Morinaga Milk Industry Co., Tokyo, Japan), the membrane was incubated overnight at 4°C with the following primary antibodies: anti-UCP1 (ab10983; Abcam, Cambridge, UK; rabbit polyclonal), anti-COX4 (A21348; Thermo Fisher Scientific, mouse monoclonal), and anti-β-actin (A1978; Sigma-Aldrich, St. Louis, MO, USA; mouse monoclonal). The bound antibodies were detected using an enhanced chemiluminescence system (Amersham, Little Chalfont, Bucks, U.K.) with horseradish peroxidase (HRP)-conjugated secondary antibodies: goat anti-rabbit immunoglobulin (Zymed Laboratories, San Francisco, CA, USA) for UCP1 and goat anti-mouse immunoglobulin (Jackson Immunoresearch Laboratories, West Grove, PA, USA) for COX4 and β-actin.

### Statistical analysis

Data are presented as the mean ± standard error. Statistical analyses were conducted using one-way analysis of variance (ANOVA), followed by the Tukey–Kramer post hoc test, with *P* < 0.05 considered significant.

## Results

### Morphological alterations during postnatal BAT development

To characterize the morphological progression of postnatal BAT development, interscapular adipose tissue was collected from hamsters on postnatal days 6–14. Gross examination demonstrated that the tissue appeared white on day 6, gradually changing from white to brownish, and exhibited a typical BAT-like appearance by day 14 (Fig. 1A). Histologically, on day 6 the depot predominantly consisted of unilocular white adipocyte, with non-adipocyte cells scattered throughout the tissue. By day 14, the tissue was primarily composed of brown adipocytes containing multilocular lipid droplets (Fig. 1B). At postnatal day 10, clusters of non-adipocyte cells with large nuclei were observed between white adipocytes; based on our previous study, these cells were considered putative progenitor-like cells associated with postnatal BAT remodeling [24]. These results indicate that postnatal BAT formation in Syrian hamsters proceeds through progressive structural remodeling process accompanied by the appearance, accumulation, and differentiation of progenitors into multilocular brown adipocytes.

**Fig. 1:**
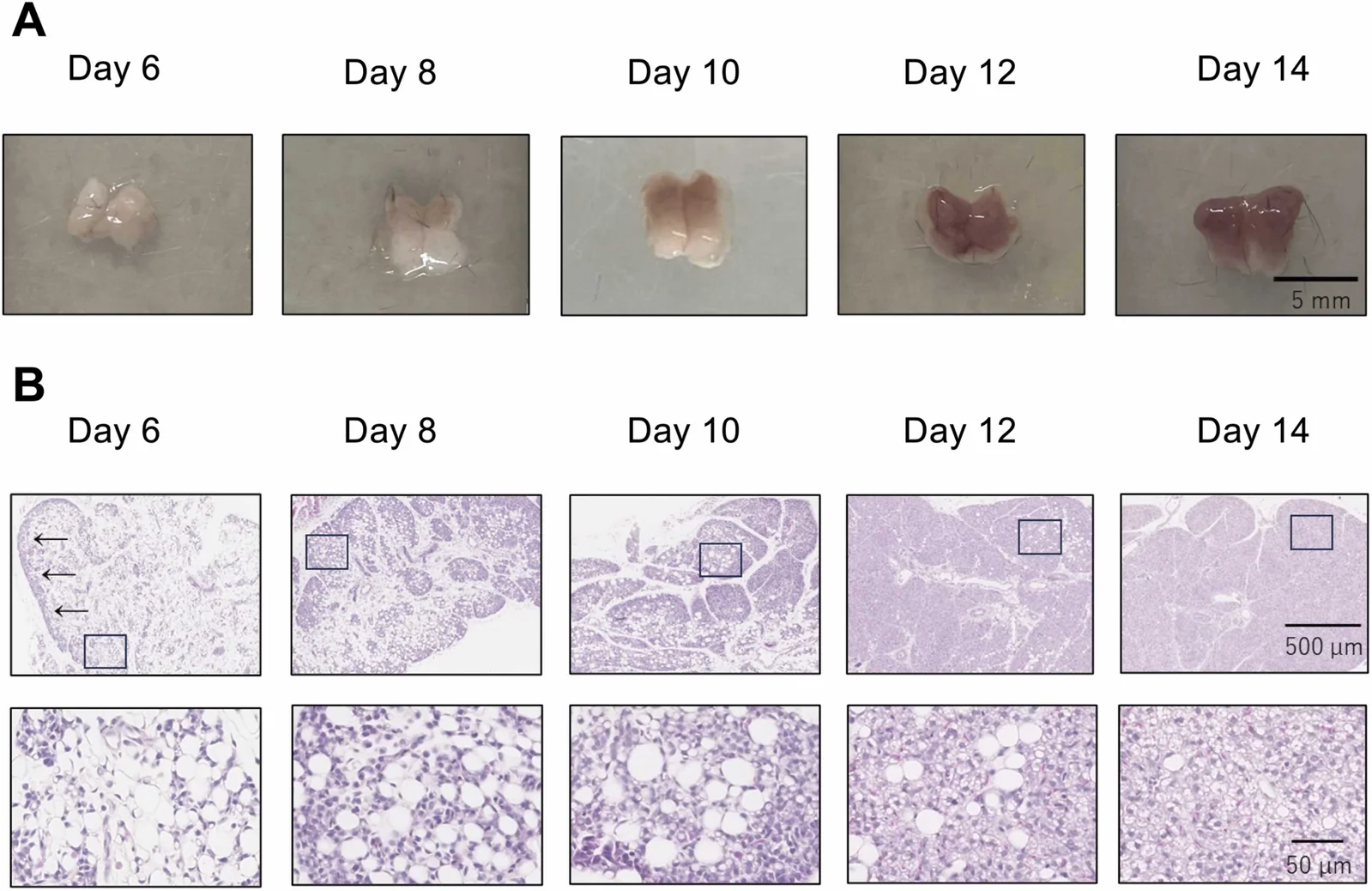
**Postnatal development of interscapular adipose tissue in Syrian hamsters.** (A) Representative macroscopic images of interscapular adipose tissue collected from hamster pups on postnatal days 6, 8, 10, 12, and 14. (B) Hematoxylin and eosin-stained sections of interscapular adipose tissue at corresponding ages. Arrows indicate regions containing progenitor-like cells. Black squares denote areas shown at higher magnification.

### Cell proliferation during postnatal BAT development

To evaluate age-dependent cell proliferation, the thymidine analog EdU (50 mg/kg), which is incorporated into newly synthesized DNA during cell proliferation, was administered intraperitoneally to pups on postnatal days 5–13. The interscapular adipose tissue was collected 24 h later and subjected to IF. MCT1-positive areas, representing progenitor-like cells and mature brown adipocytes, were sparsely distributed on postnatal day 6 but progressively expanded with age, occupying most of the tissue by day 14 (Fig. 2A). The MCT1-positive area was quantified and expressed as a percentage of the total tissue area. At 6 days of age, the value was approximately 9% of total tissue area and increased with age (Fig. 2B). The rate of increase in MCT1-positive area was highest between postnatal days 8 and 10 (48.3% ± 3.5%), indicating that progenitor-derived cells expanded most prominently during this period (Fig. 2C). The percentage of EdU-positive cells was 27.1% ± 1.5% in 6 days, peaked at 38.0% ± 1.6% in 8 days, and decreased to 16.5% or below thereafter. These results indicate that overall cellular proliferative activity was highest at postnatal day 8 (Fig. 2D).

**Fig. 2:**
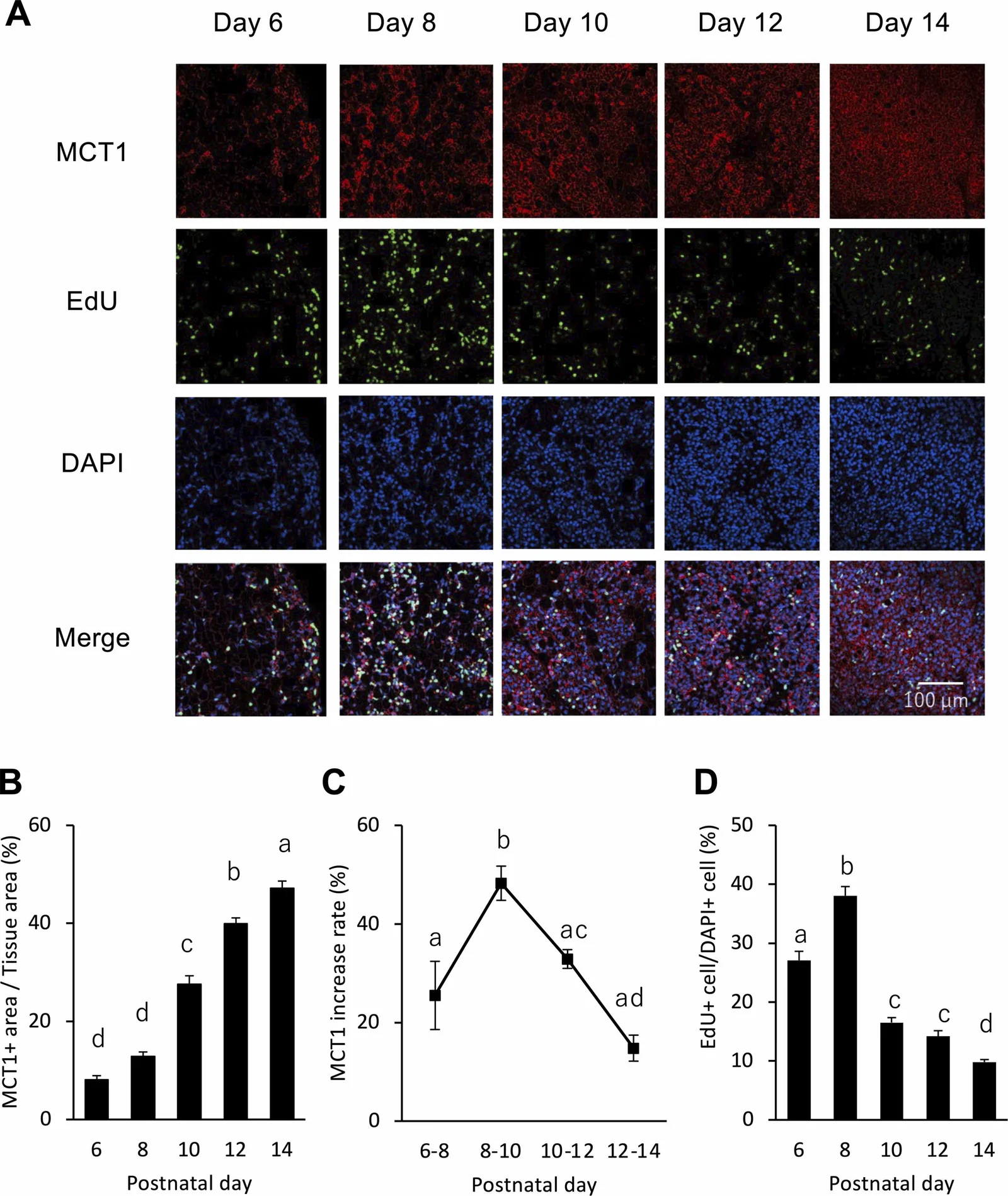
**Distribution of progenitors during postnatal development of interscapular adipose tissue.** 5-ethynyl-2′-deoxyuridine (EdU; 50 mg/kg) was administered intraperitoneally to pups on postnatal days 5–13, and tissues were collected 24 h later for analysis. (A) Representative immunofluorescence images of interscapular adipose tissue sections. Nuclei were stained with 4′,6-diamidino-2-phenylindole (DAPI; blue). Monocarboxylate transporter 1 (MCT1; red) was used to identify progenitor-like cells and mature brown adipocytes, and EdU (green) labels the nuclei of proliferating cells. (B) Quantification of the MCT1-positive area relative to the total tissue area. (C) Rate of increase in MCT1-positive area calculated from the data shown in B. For example, for 8-day-olds, {(individual data at 8 days old) − (average value of data at 6 days old)} ÷ (individual data at 8 days old) × 100. (D) Percentage of EdU-positive cells in tissue sections. Data are presented as the mean ± SEM (n = 4). Different letters indicate significant differences among ages (P < 0.05).

### Temporal pattern of sympathetic innervation in postnatal BAT

To examine the temporal pattern of sympathetic innervation during postnatal development, interscapular adipose tissue sections from postnatal days 6–14 were subjected to IF staining for TH, a marker of sympathetic nerves. Sympathetic nerves were undetectable at 6 days of age and were found sporadically throughout the tissue after 8 days of age (Fig. 3A). The TH-positive area was quantified as a percentage of the total tissue area. At postnatal day 8, the TH-positive area was comparable to that observed at subsequent postnatal stages and remained relatively constant throughout the observation period (Fig. 3B). The rate of increase in TH-positive area was highest between postnatal days 6 and 8 (98.4% ± 0.5%) and remained low (<10%) thereafter (Fig. 3C).

**Fig. 3:**
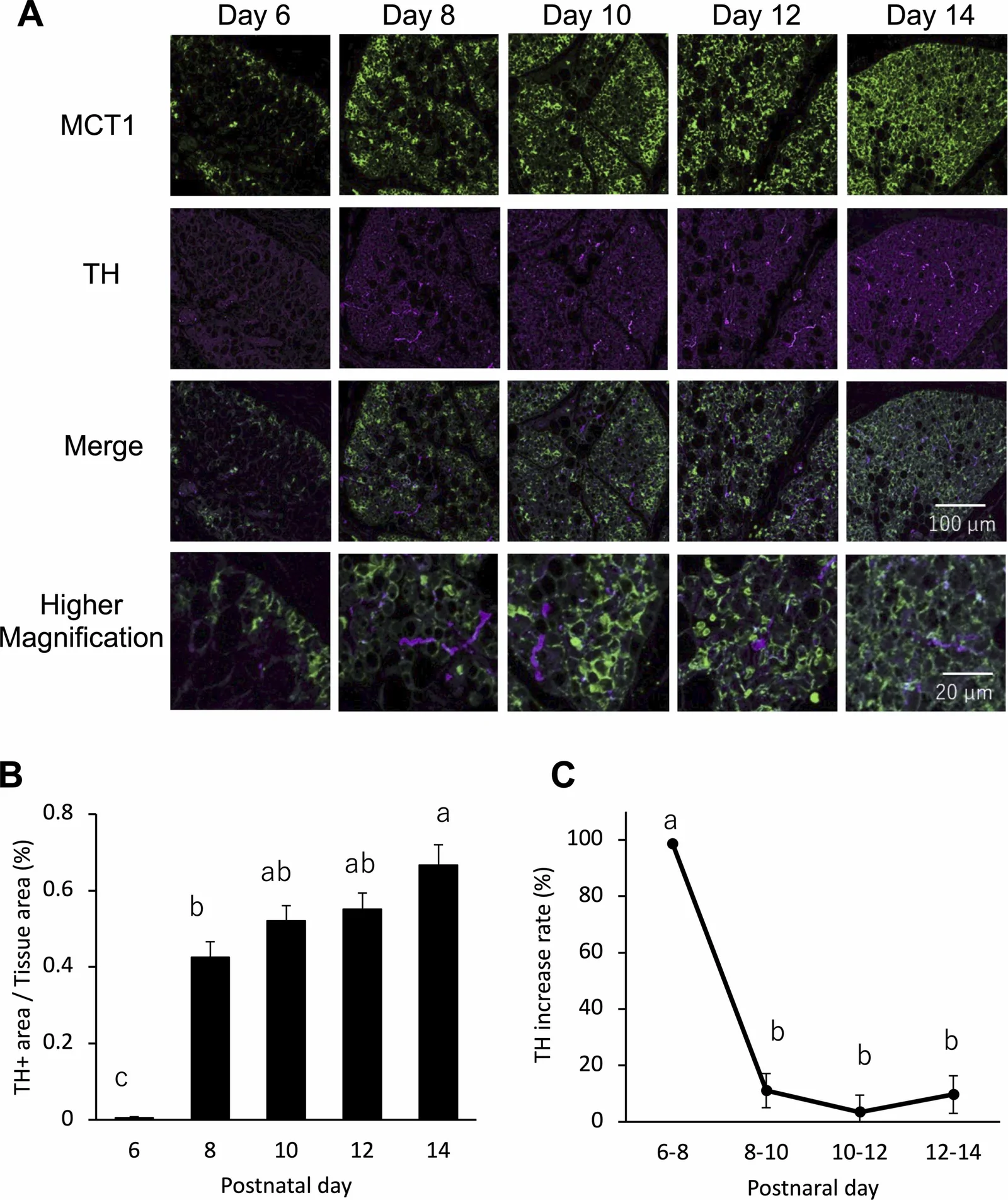
**Localization of sympathetic innervation in interscapular adipose tissue.** (A) Representative immunofluorescence images of interscapular adipose tissue sections from pups on postnatal days 6, 8, 10, 12, and 14. Monocarboxylate transporter 1 (MCT1; green) was used to identify progenitor-like cells and mature brown adipocytes, and tyrosine hydroxylase (TH; purple) was used to detect sympathetic nerve fibers. (B) Quantification of the TH-positive area relative to the total tissue area. (C) Rate of increase in the TH-positive area calculated from the data shown in B. For example, for an 8-day-old, {(individual data at 8 days) − (average data at 6 days)} ÷ (individual data at 8 days) × 100. Data are presented as the mean ± SEM (n = 4). Different letters indicate significant differences among ages (P < 0.05).

### Postnatal changes in vascular volume in BAT

To examine the temporal pattern of vascular development during postnatal BAT development, interscapular adipose tissue sections from 6 to 14-day-old hamster pups were subjected to IF staining using MAM-lectin to visualize vascular endothelial cells. Vascular endothelial cells stained with MAM-lectin were scattered throughout the tissue on postnatal day 6 and increased with age (Fig. 4A). The percentage of MAM-lectin–positive area relative to the total tissue area was quantified. At 6 days of age, the MAM-lectin-positive area was 2% and gradually increased through postnatal development (Fig. 4B). The rate of increase in MAM-lectin–positive area was 24.9% ± 4.9% between postnatal days 6 and 8 and 34.8% ± 4.8% between postnatal days 8 and 10, suggesting that vascular development occurs concurrently with BAT remodeling during postnatal development (Fig. 4C).

**Fig. 4:**
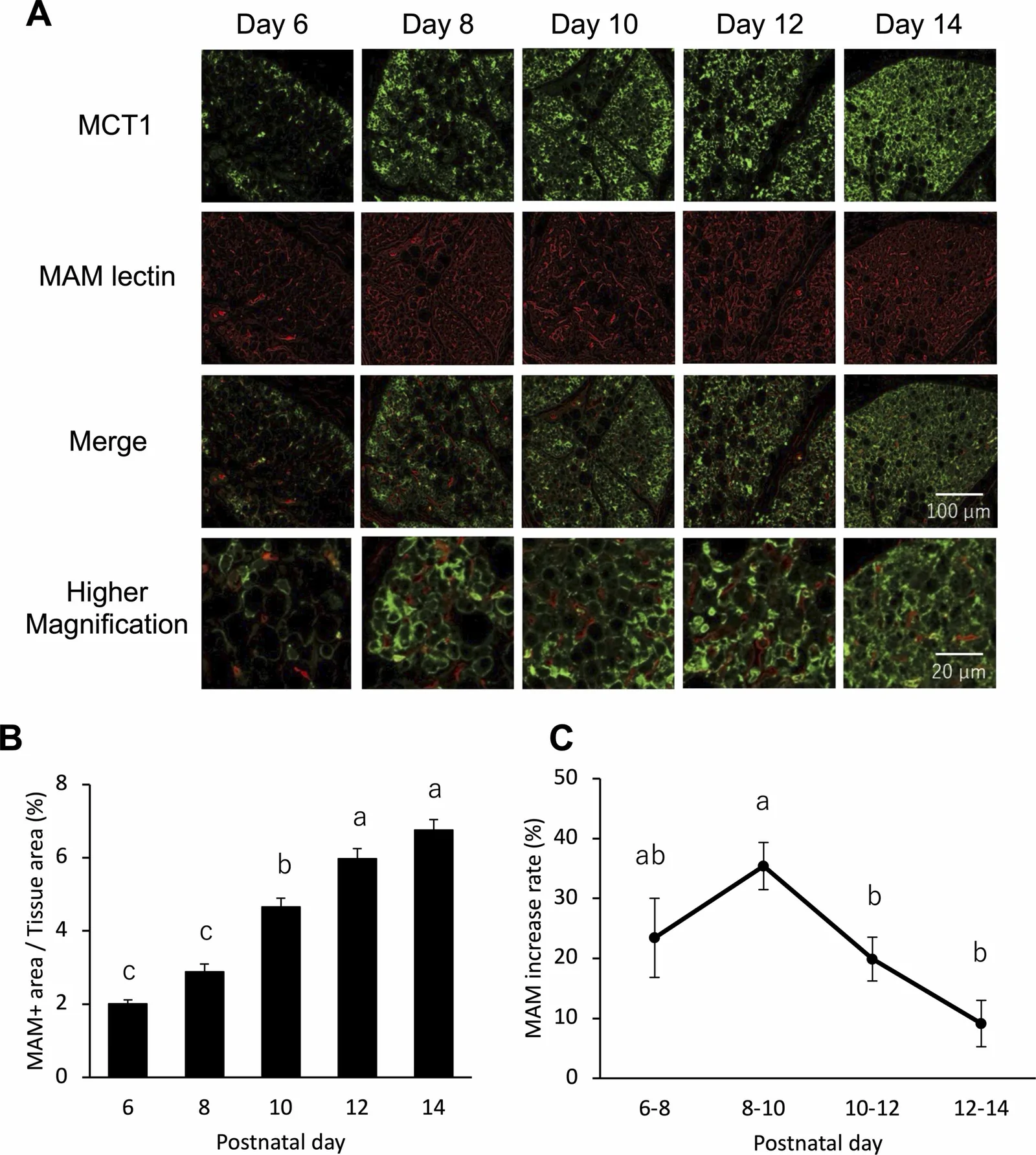
**Localization of vasculature in interscapular adipose tissue.** (A) Representative immunofluorescence images of interscapular adipose tissue sections from pups on postnatal days 6, 8, 10, 12, and 14. Monocarboxylate transporter 1 (MCT1; green) was used to identify progenitor-like cells and mature brown adipocytes, and *Maackia amurensis* agglutinin (MAM-lectin; red), was used to visualize blood vessels. (B) Quantification of the MAM lectin-positive area relative to the total tissue area. (C) Rate of increase in the MAM-positive area calculated from the data shown in B. For example, for 8-day-olds, {(individual data at 8 days old) − (average value of data at 6 days old)} ÷ (individual data at 8 days old) × 100. Different letters indicate significant differences among ages (P < 0.05).

### Effects of VEGFR inhibition on postnatal BAT development and molecular markers

To investigate the role of angiogenesis in postnatal BAT development, hamster pups were administered SU5416, a selective VEGFR2 inhibitor. Pups were divided into control, low-dose, and high-dose groups. Each group received daily intraperitoneal injections of DMSO, SU5416 (4 mg/kg), or SU5416 (15 mg/kg) from postnatal days 5–9. Interscapular adipose tissue was harvested on postnatal day 10 (Fig. 5A). The body weight of the hamsters at 10 days of age was not significantly different among the groups (Fig. 5B). The weight of interscapular adipose tissue tended to decrease in the low-concentration group and significantly decreased in the high-concentration group compared with control (Fig. 5C). Macroscopic examination revealed that in the control and low-dose groups, the cranial portion exhibited a brownish color while the caudal portion was white, whereas interscapular adipose tissue from high-dose group appeared uniformly white throughout the tissue. Histological analysis revealed a mixture of progenitor-like cells and white adipocytes throughout the tissue in all groups, with a high proportion of progenitor-like cells. The control and low-concentration groups exhibited scattered progenitor-like cells, whereas the high-concentration group had fewer progenitor-like cells and a predominance of white adipocytes (Fig. 5D).

**Fig. 5:**
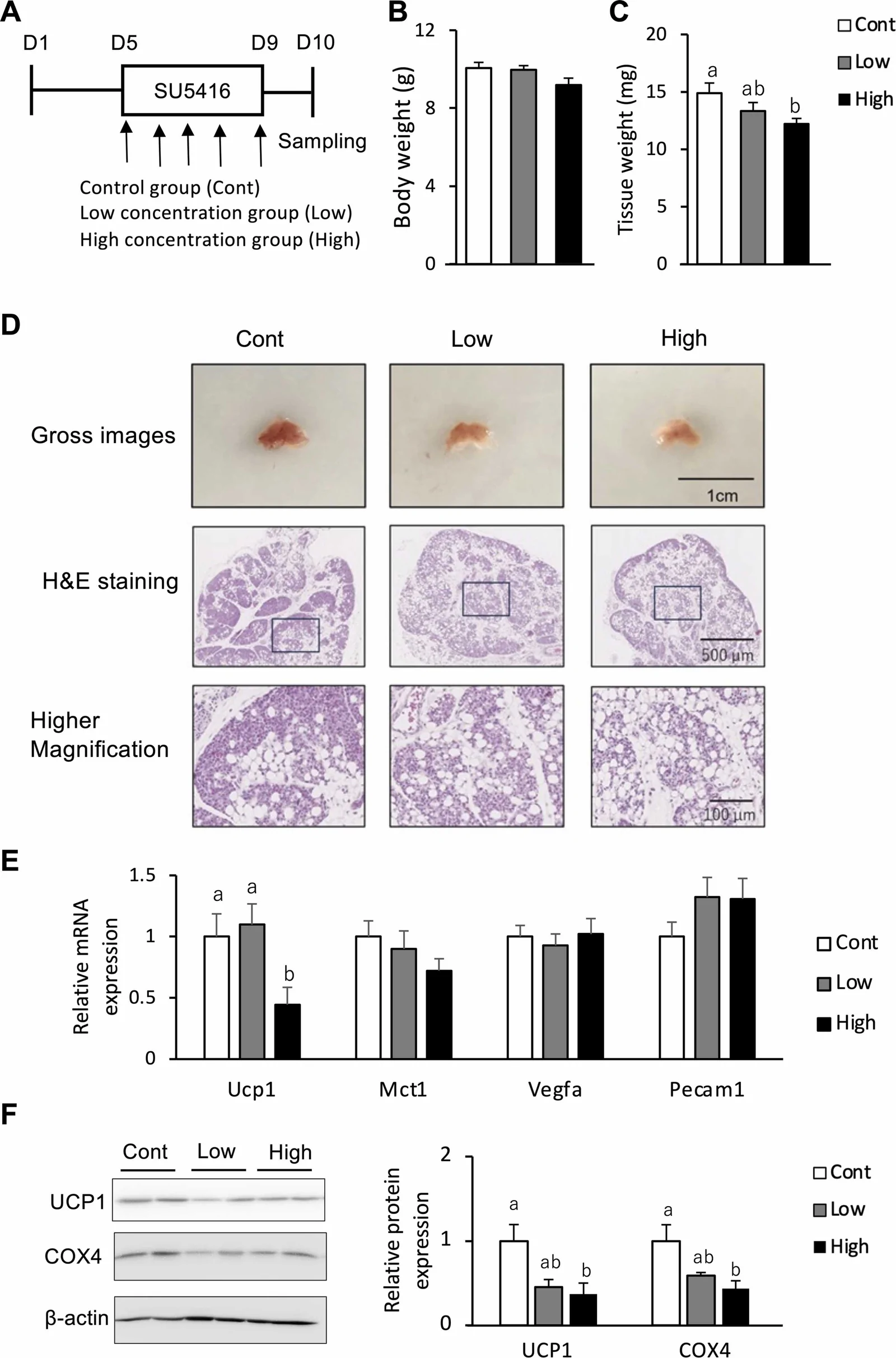
**Effects of VEGFR inhibition on postnatal development and molecular markers of interscapular adipose tissue.** (A) Experimental design. SU5416, a vascular endothelial growth factor receptor (VEGFR) inhibitor, was administered intraperitoneally once daily to hamsters from postnatal day 5 to day 9, and interscapular adipose tissue was collected on postnatal day 10. Pups were divided into control (Cont, DMSO), low-dose (Low, 4 mg/kg), and high-dose (High, 15 mg/kg) groups. (B) Body weight and (C) interscapular adipose tissue weight of hamsters at 10 days of age. (D) Representative macroscopic images of interscapular adipose tissue and hematoxylin and eosin (H&E)-stained histological sections. Black squares indicate the regions shown at higher magnification. (E) Relative mRNA expression levels of *Ucp1*, *Mct1*, *Vegfa*, and *Pecam1* in interscapular adipose tissue. mRNA levels were normalized to *Actb* and expressed relative to the control group. (F) Representative immunoblots of UCP1 and COX4 protein expression. Protein levels were normalized to Actb. Data are presented as means ± SEM (n = 9 for control and low-dose groups; n = 10 for the high-dose group for mRNA analysis; n = 4 for control and high-dose groups and n = 3 for the low-dose group for protein analysis). Different letters indicate significant differences among groups (P < 0.05).

To assess the effect of VEGFR inhibition on molecular markers of BAT, both gene and protein expression analyses were performed. *Ucp1* mRNA expression was significantly reduced to 44.5% of control levels in the high-concentration group, whereas no significant change was observed in the low-concentration group (Fig. 5E). *Mct1* mRNA showed a dose-dependent reduction trend, although this difference was not statistically significant. The mRNA expression levels of *Vegfa and Pecam1* (platelet endothelial cell adhesion molecule 1), an endothelial cell marker, were not significantly different among groups. Consistent with the mRNA results, UCP1 and COX4 protein levels were significantly reduced in the high-dose group compared with control. To examine whether the effects of SU5416 could be attributed to off-target inhibition of KIT or MET, pups were treated with the KIT inhibitor imatinib or the MET inhibitor capmatinib. Neither inhibitor reproduced the reduction in *Ucp1* expression observed following SU5416 treatment; imatinib had no significant effect on *Ucp1* expression, whereas capmatinib increased *Ucp1* expression (Supplementary Fig. S1).

Collectively, these findings indicate that SU5416 treatment attenuated postnatal BAT development and suppressed thermogenic marker expression, suggesting that VEGFR-dependent signaling may contribute to the accumulation of progenitor-like cells and brown adipocyte maturation during early postnatal development.

### Effects of VEGFR inhibition on vascular volume and cell proliferation in postnatal BAT

To assess the effects of VEGFR inhibition on vascular abundance, vascular volume was quantified following SU5416 administration. IF staining revealed that areas stained with MCT1 (green) were scattered throughout the tissue in all groups (Fig. 6A). The relative MCT1-positive areas were unchanged in the low-concentration group (0.99 ± 0.07) and tended to decrease in the high-concentration group (0.76 ± 0.07), however no significant differences were observed among groups (Fig. 6B). Vascular endothelial cells stained with MAM-lectin were observed throughout the tissue in all groups, and the percentage of MAM-lectin-positive areas relative to tissue area did not differ significantly among groups (Fig. 6C).

**Fig. 6:**
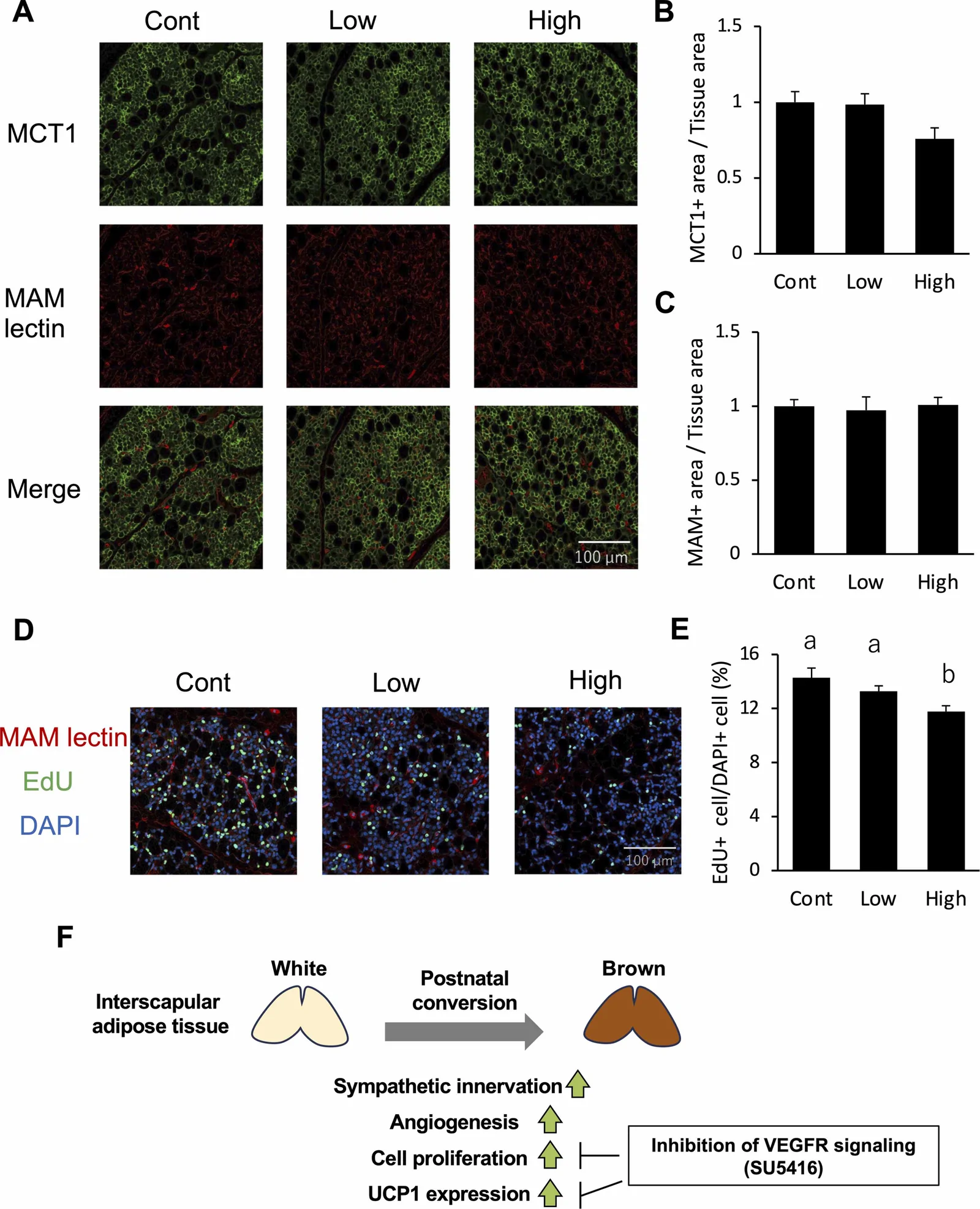
**Effect of VEGFR inhibition on progenitor and vascular abundance and cell proliferation in the interscapular adipose tissue.** Hamster pups were treated with SU5416 as described in Fig. 5, and interscapular adipose tissue was collected on postnatal day 10. Immunofluorescence staining was performed on tissue sections. (A-C) Immunofluorescence analysis of progenitor and vascular abundance. (A) Representative images showing MCT1 (green), a marker of progenitor-like cells and mature brown adipocytes, and *Maackia amurensis* agglutinin (MAM-lectin) (red), which labels glycans enriched on vascular endothelial cells and marks blood vessels. (B) Quantification of the MCT1-positive area relative to the total tissue area. (C) Quantification of the MAM lectin–positive area relative to the total tissue area. (D-E) Analysis of cell proliferation. EdU was administered at the time of the final SU5416 injection, and tissue was collected 24 h later. (D) Representative images showing DAPI (blue) for nuclei, MAM lectin (red) for blood vessels, and EdU (green) for proliferating cells. (E) Quantification of the percentage of EdU-positive cells. Data are presented as means ± SEM (n = 5). Different letters indicate significant differences among groups (P < 0.05). (F) Schematic summary of the findings of the present study.

To examine the effect of VEGFR inhibition on cell proliferation in the interscapular adipose tissue, EdU (50 mg/kg) was administered intraperitoneally at the time of the final SU5416 injection, and tissues were collected 24 h later. EdU incorporation was used to identify cells undergoing DNA synthesis during the labeling period. IF staining of interscapular adipose tissue sections (Fig. 6D) revealed that the proportion of EdU-positive cells relative to cell nuclei (DAPI) was 14.5% ± 0.7% in the control group. This proportion decreased to 13.3% ± 0.4% in the low-dose group and was significantly reduced to 11.8% ± 0.4% in the high-dose group (Fig. 6E). The number of EdU-positive cells was reduced following SU5416 treatment, suggesting reduced overall proliferative activity. Among EdU-positive cells, only a small fraction was MAM lectin-positive endothelial cells, and this proportion did not differ among groups.

Collectively, these results suggest that VEGFR-dependent signaling may contribute to postnatal BAT formation without markedly affecting overall vascular abundance, as assessed by MAM lectin staining, under the present experimental conditions (Fig. 6F).

## Discussion

In Syrian hamsters, BAT forms postnatally through a progressive conversion from WAT, involving progenitor proliferation, gradual replacement of white adipocytes, and subsequent differentiation into mature brown adipocytes [24]. In this study, we examined the respective contributions of sympathetic nerves and blood vessels in postnatal BAT formation. Importantly, Syrian hamsters represent a valuable experimental model for investigating the mechanisms underlying BAT development. In many mammalian species, BAT is largely formed during the fetal period, making it challenging to distinguish intrinsic developmental programs from maternal influences. In contrast, BAT in hamsters develops postnatally. This allows researchers to exclude maternal effects and directly evaluate the roles of endogenous regulatory mechanisms and environmental inputs, as well as to dissect the molecular mechanisms that govern BAT formation and maturation.

In the present study, sympathetic innervation was undetectable at postnatal day 6 but was observed from day 8, after which the TH-positive area remained relatively constant. Previous studies in mice have demonstrated that the thermogenic identity and basal BAT activity are established during late embryogenesis and early postnatal development largely independent of sympathetic input, highlighting the importance of intrinsic cellular programs in BAT development [26]. In contrast, once BAT identity is established, its long-term maintenance and adaptive thermogenic activation depend on distinct sympathetic pathways that innervate both parenchymal and vascular compartments [14], [15], [16]. However, these observations may appear to contradict our previous report showing that BAT development is suppressed under warm environmental conditions. Under warm environmental conditions, where sympathetic nerve activity is expected to be low, reduced UCP1 expression, a diminished UCP1-positive area, and decreased progenitor proliferation were observed in the interscapular depot [25]. However, it should be noted that BAT development still proceeded under warm conditions: BAT formation was delayed rather than abolished [25]. Thus, the SNS may regulate the speed of postnatal BAT formation rather than the developmental process itself. However, because our analysis was primarily morphological, a functional contribution of sympathetic signaling cannot be excluded.

We further evaluated the role of the vasculature in BAT development. Blood vessels were distributed throughout the tissue from early postnatal stages and increased during postnatal development. We previously observed that reduced vascular density and decreased VEGFA expression were accompanied by suppressed progenitor proliferation in hamsters raised at warm temperatures [25]. Consistent with this, treatment with the VEGFR inhibitor SU5416 reduced UCP1 expression and progenitor-like cell accumulation. However, unexpectedly, blood vessel volume was not significantly altered following SU5416 treatment, indicating that inhibition of VEGFR signaling impaired BAT formation without inducing overt regression of established vascular structures. Although SU5416 is widely used as an angiogenesis inhibitor, its primary mechanism is the blockade of VEGFR2 tyrosine kinase signaling rather than the immediate elimination of pre-existing vessels [27], [28]. Indeed, VEGF signaling is essential for angiogenic growth and endothelial survival in immature vessels but is less critical for the maintenance of stable, mature vasculature [29], [30]. Thus, suppression of endothelial VEGFR signaling may disrupt paracrine communication within the vascular niche without markedly affecting gross vascular volume. Emerging evidence indicates that the vascular niche plays an active role in regulating adipocyte lineage dynamics. In mice, UCP1-expressing adipocytes are enriched in perivascular regions, and mural or vessel wall-associated progenitor cells have been shown to contribute to thermogenic adipocyte formation [31]. Together, these findings suggest that VEGFR-dependent signaling, rather than overall vascular abundance itself, may contribute to the accumulation of progenitor-like cells. However, the cellular source of VEGF was not determined in the present study. In mice, endothelial VEGFR2 signaling promotes endothelial secretion of PDGF-CC, which acts on PDGFRα-positive progenitors of beige adipocytes, a brown adipocyte-like cell population induced within white adipose tissue [32]. In addition, VEGFA expression is increased in the stromal vascular fraction (SVF) during beige adipogenesis [33], suggesting that SVF cells, including endothelial cells, adipocyte progenitors, pericytes, and fibroblasts, could contribute to VEGF production. Whether similar paracrine interactions occur during postnatal BAT development in Syrian hamsters remains unknown and warrants further investigation.

An additional explanation for the effects of SU5416 is its inhibitory activity against receptor tyrosine kinases (RTKs) other than VEGFR. SU5416 is known to inhibit multiple RTKs, including KIT, MET, FLT3, and RET. Although direct evidence linking these kinases to progenitor proliferation remains limited, c-Kit signaling has been implicated in brown adipocyte lipid accumulation, and MET inhibition suppresses preadipocyte differentiation [34], [35]. More broadly, RTK signaling regulates cell proliferation, survival, and differentiation. Adipocyte differentiation is initiated by C/EBPβ, followed by sequential activation of C/EBPα and PPARγ, which together drive the transcriptional program required for adipocyte maturation [36], [37]. Thus, disruption of RTK signaling may impair both progenitor expansion and activation of the adipogenic gene network. However, neither the selective KIT inhibitor imatinib nor the selective MET inhibitor capmatinib reproduced the SU5416-induced molecular phenotype, including reduced *Ucp1* mRNA expression, suggesting that inhibition of KIT or MET alone is unlikely to fully account for the effect of SU5416. Nevertheless, the contribution of other off-target effects of SU5416 cannot be excluded. Collectively, these observations suggest that SU5416-induced suppression of progenitor proliferation may reflect inhibition of endothelial signaling or off-target RTK pathways rather than blockade of angiogenesis alone. Further studies using more selective genetic or complementary pharmacological approaches will be necessary to identify the specific signaling pathways affected by SU5416 and to determine the relative contributions of vascular development and growth factor-mediated progenitor regulation during postnatal BAT formation.

## Conclusions

In conclusion, this study demonstrates that postnatal BAT formation in Syrian hamsters is accompanied by coordinated vascular development and progenitor-like cell expansion, which requires signaling through VEGF receptors or other tyrosine kinase receptors inhibited by SU5416. Further mechanistic studies are required to define the precise signaling pathways linking vascular niches, progenitor dynamics, and functional maturation in developing BAT. These findings provide new insight into the cellular and molecular mechanisms governing BAT development. Syrian hamsters, in which BAT develops postnatally are a useful model for elucidating the mechanisms underlying BAT development.

## CRediT authorship contribution statement

**Khaled Mahmud Sujan:** Writing – original draft, Visualization, Methodology, Investigation, Formal analysis, Data curation. **Yukino Tamakoshi:** Writing – review & editing, Investigation, Formal analysis, Data curation. **Bong Soo Seok:** Writing – review & editing, Investigation, Data curation. **Zibo Yang:** Writing – review & editing, Validation. **Shucheng Xu:** Writing – review & editing, Validation. **Mohamed Elfeky:** Writing – review & editing. **Mira Kato-Suzuki:** Writing – review & editing. **Yuko Okamatsu-Ogura:** Writing – review & editing, Supervision, Project administration, Funding acquisition, Formal analysis, Conceptualization.

## Ethics declaration

This study was conducted in accordance with the ARRIVE (Animal Research: Reporting of In Vivo Experiments) guidelines. This study was approved by the Hokkaido University. (Approval Nos. 18–0052 and 22–0116)

## Funding

This work was supported by the Japan Society for the Promotion of Science (10.13039/501100001691JSPS) KAKENHI (Grant Numbers 22K08641, 23H04942, 23H04939, and 25K22770), the Naito Foundation, and the Akiyama Life Science Foundation.

## Declaration of Competing Interest

The authors declare that they have no known competing financial interests or personal relationships that could have appeared to influence the work reported in this paper.

## Data Availability

All data supporting the findings of this study are available from the corresponding author upon request.

## Footnotes

Appendix A Supplementary data associated with this article can be found in the online version at doi:10.1016/j.jphyss.2026.100106.
